# Discordance between dual-energy X-ray absorptiometry bone mineral density and spinal computed tomography texture analysis: An investigation into low correlation rates

**DOI:** 10.1016/j.afos.2024.01.002

**Published:** 2024-03-01

**Authors:** Min Woo Kim, Young Min Noh, Jung Wook Huh, Han Eol Seo, Dong Ha Lee

**Affiliations:** Department of Orthopedic Surgery, Busan Medical Center, Busan, South Korea

**Keywords:** Dual-energy X-ray absorptiometry (DXA), Computed tomography hounsfield unit (CT HU), Bone mineral density (BMD), Morphometric texture analysis, Linear regression

## Abstract

**Objectives:**

This research delves into the application of texture analysis in spine computed tomography (CT) scans and its correlation with bone mineral density (BMD), as determined by dual-energy X-ray absorptiometry (DXA). It specifically addresses the discordance between the 2 measurements, suggesting that certain spinal-specific factors may contribute to this discrepancy.

**Methods:**

The study involved 405 cases from a single institution collected between May 6, 2012 and June 30, 2021. Each case underwent a spinal CT scan and a DXA scan. BMD values at the lumbar region (T12 to S1) and total hip were recorded. Texture features from axial cuts of T12 to S1 vertebrae were extracted using gray-level co-occurrence matrices, and a regression model was constructed to predict the BMD values.

**Results:**

The correlation between CT texture analysis results and BMD from DXA was moderate, with a correlation coefficient ranging between 0.4 and 0.5. This discordance was examined in light of factors unique to the spine region, such as abdominal obesity, aortic calcification, and lumbar degenerative changes, which could potentially affect BMD measurements.

**Conclusions:**

Emerging from this study is a novel insight into the discordance between spinal CT texture analysis and DXA-derived BMD measurements, highlighting the unique influence of spinal attributes. This revelation calls into question the exclusive reliance on DXA scans for BMD assessment, particularly in scenarios where DXA scanning may not be feasible or accurate.

## 1 Introduction

Osteoporosis, a prevalent bone disease, is characterized by a reduction in bone mass and strength, leading to an increased risk of fractures [1]. This risk is notably high in areas abundant in trabecular bone, such as the proximal femur, vertebral body of the spine, and the distal radius. The complexity of the disease underscores the need for a robust diagnostic system for its effective management.

The central dual-energy X-ray absorptiometry (DXA) of the lumbar vertebrae and femoral bone currently serves as the reference standard for diagnosing osteoporosis [2]. However, DXA measurements can become complex due to inherent variability, influenced by factors like the progression of osteosclerosis and the degree of adiposity [3]. This complexity is particularly noticeable in the lumbar spine, where measurements can be overestimated due to scoliosis, degenerative arthritis, bone tissue formation, osteosclerosis, and subcutaneous fat [4].

Despite the DXA T-score being endorsed by the World Health Organization (WHO) as the standard for osteoporosis diagnosis, it does have limitations, particularly when compromised by degenerative changes. In this study, we propose a novel approach, extending the commonly used region of interest (ROI) from L1-L4 to T12-S1 in spine CT scans. This approach aims to provide a more comprehensive understanding of bone health. Although our initial goal was to explore the correlation between BMD as measured by DXA and texture analysis values derived from these CT scans, the correlation was not as strong as expected.

Recognizing this, we shifted our focus to developing a method akin to the trabecular bone score (TBS) [5], but distinct in its use of the gray level co-occurrence matrix (GLCM) [6,7]. With GLCM, we extract 45 different texture analysis values from the CT scans, which are then processed through machine learning techniques to establish correlations and create a predictive model. Our comprehensive data set, including follow-up patients, provides unique insights into the temporal progression of bone health.

Our findings underscore the potential of texture analysis as a reliable and efficient tool for monitoring bone mineral status, offering an opportunity for more precise diagnosis and management of osteoporosis. Notably, this study represents a significant advancement in the field by extending the typical ROI used in DXA, thereby enhancing the assessment of osteoporosis, and overcoming limitations in the traditional approach.

## 2 Methods

Our retrospective study, approved by the institutional review board (2023-01-001), initially involved 1720 cases from 843 patients. All patients underwent both spine CT and DXA scans at the same institution between May 9, 2011 and July 30, 2022 for the evaluation of spine-related diseases, including the differentiation of spine pathologies and assessment of fractures. The spine CT scans were integral in providing detailed imagery for the diagnosis and management of these conditions.

A meticulous selection process was then conducted, which led to a refined cohort comprising 405 cases from 219 patients. This selection was based on the adherence to a specific criterion—a temporal gap of less than a month between the spine CT and DXA scan dates. The rationale behind this criterion was to ensure a close temporal correlation between the 2 diagnostic modalities, thereby enhancing the reliability of our findings in understanding the relationship between spine CT results and DXA measurements in a real-world clinical context.

During the subsequent selection process, cases were excluded if they met any of the following conditions: absence of an actual measurable axial cut from T12 to S1 vertebrae in the CT images; historical instances of compression fractures or burst fractures anywhere from T12 to S1; previous surgical interventions due to fractures, including vertebroplasty or kyphoplasty for spinal compression fractures; or the existence of metal artifacts from unstable burst fractures; or difficulties in identifying trabecular bones due to severe osteolytic or pathological changes.

**Fig. 1:**
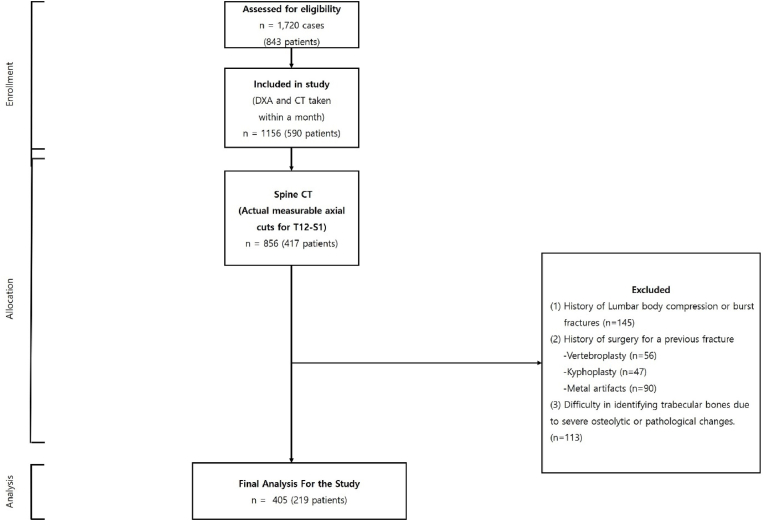
Flowchart illustrating the selection process of patients undergoing concurrent spine CT and DXA scans. CT, computed tomography; DXA, dual-energy X-ray absorptiometry.

This exclusion process resulted in a final cohort comprising 405 cases from 219 patients who were included in our analysis (Fig. 1). By conducting a rigorous selection process, we ensured the robustness and reliability of our study, focusing on the most relevant and insightful cases for our investigation into the potential of texture analysis in monitoring bone mineral status. By selecting an extended region of interest from T12 to S1, this study provides a more comprehensive approach to diagnosing and managing osteoporosis.

### 2.1 *CT and DXA imaging protocols*

The CT scans were carried out using a Siemens SOMATOM 128, Definition AS + scanner (Siemens Healthcare, Forchheim, Germany). The standardized protocol for each scan entailed a single-energy CT scan with settings at 120 kVp and 247 mA, featuring a dose modulation with a 0.6 mm collimation. The effective pitch was maintained at 0.8, and the reconstruction kernel employed was B60 (sharp). For the spine CT scans, which were executed without the use of contrast, a reconstructed slice thickness of 5.0 mm was consistently preserved.

For the DXA scans, a standard device was utilized, adhering to a conventional protocol (GE Lunar Prodigy, GE Healthcare). The subsequent reports were produced using vendor-specific software (Physicians Report Writer DX; Hologic, Discovery WI, USA). The strict adherence to standardized imaging protocols throughout the study guarantees the reproducibility and uniformity of our results.

### 2.2 *Regions of interest*

**Fig. 2:**
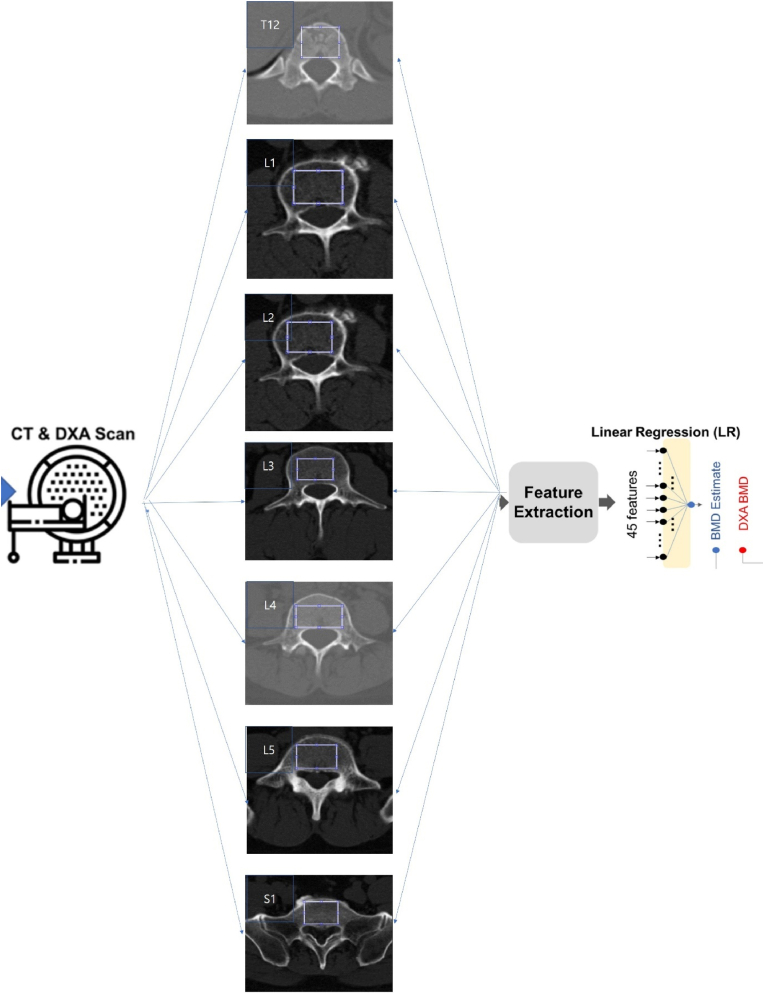
Schematic flow for BMC and BMD estimations from computed tomography. BMC, bone mineral content; BMD, bone mineral density.

The regions of interest (ROIs) for statistical measurement from bone images were limited to the trabecular part of the bone to prevent distortion in measurements. Among various methods for isolating ROIs, we chose the thresholding method [8] for this research. For each patient, a 2-dimensional (2D) slice image was selected from the spine CT axial cut, where the 2D image encompassed the maximum axial trabecular area of the vertebral bodies between T12 and S1. As shown in Fig. 2, our texture analysis was conducted within a rectangular region that covered most of the trabecular area. This method allowed for a comprehensive evaluation across the extended ROI, providing greater depth to our findings.

### 2.3 *Feature extraction*

In our study, we extracted a total of 45 features from the designated ROIs. This included 5 intensity-based features obtained through histogram analysis and 40 texture-based features sourced from a Gray Level Co-occurrence Matrix (GLCM). The intensity-based features—mean, standard deviation, skewness, kurtosis, and entropy—were garnered from the histogram of the ROI image. These fundamental features help interpret various bone intensity characteristics such as brightness, asymmetry, randomness, uniformity, and sharpness.

Concurrently, the texture-based features provided insights into the spatial relationships between adjacent pixels on the 2D image. They were extracted from the GLCM, which is a matrix representation of the frequency of occurrence of pixel intensity values in an image. In this study, we constructed 8 GLCMs for each sample ROI image at 4 levels (N = 16, 32, 64, 128) and 2 directions (horizontal and vertical). From each GLCM, we derived 5 statistics: entropy, contrast, correlation, homogeneity, and variance.

These 45 features were then incorporated into a linear regression (LR) model and an artificial neural network (ANN) model. The LR model predicted BMD using a linear combination of the 45 input features. The ANN model, a fully connected neural network, consisted of 3 hidden layers with 8 nodes in the first and second layers and 2 nodes in the third layer, using a rectified linear unit as the non-linear operator (as depicted in Fig. 2).

### 2.4 *Correlation assessment*

In our study, we utilized either a LR model or a fully connected ANN to predict BMD and analyze the correlation between the predicted values and the reference DXA BMD values.

During preprocessing, each feature value was normalized using the sample mean and standard deviation. Similarly, every BMD reference was also normalized. In the LR model, BMD was estimated as a weighted sum of the 45 normalized features and a bias term. The weights in this model were determined by minimizing the mean squared error (MSE), which represents the difference between the predicted and reference BMD values.

Given that the number of samples in our dataset was greater than the number of trainable weights, overfitting was not a concern in this study. Therefore, we did not divide the dataset into training and testing subsets, nor did we use any regularization techniques such as ridge regression or the least absolute shrinkage and selection operator (LASSO).

## 3 Results

### 3.1 *Patient demographics*

Our study encompassed a total of 405 cases from 219 patients, with 105 men and 114 women participating. The average age and BMI of the participants were 58.12 ± 9.26 years and 24.19 ± 4.65 kg/m^2^, respectively. The average time interval between the spinal CT and DXA was 2.89 ± 5.22 days (Table 1.). Finally, the baseline diagnosis of osteoporosis, osteopenia, and normal BMD of the enrolled participants are shown in Table 2.

**Table 1:** Demographic and clinical characteristics of the study participants.

Cases (Subjects)	405 (219)
Age, yrs	58.12 ± 9.26
The time between CT and DXA dates, days	2.89 ± 5.22
Sex, male/female	105/114
BMI, kg/m^2^	24.19 ± 4.65

Values are expressed as mean ± standard deviation.

CT, computed tomography; DXA, dual-energy X-ray abostroptiometry; BMI, body mass index.

**Table 2:** The baseline diagnosis of the osteoporosis, osteopenia and normal BMD of the enrolled participants.

Age, yrs	Normal	Osteopenia	Osteoporosis	BMD, g/cm^2^
Lumbar spine				
21–30	6 (60.0)	4 (40.0)	0 (0.0)	0.958 ± 0.112
31–40	25 (47.2)	22 (41.5)	6 (11.3)	0.967 ± 0.133
41–50	27 (54.0)	20 (40.0)	3 (6.0)	0.957 ± 0.121
51–60	25 (32.1)	33 (42.3)	20 (25.6)	0.872 ± 0.153
61–70	8 (28.6)	12 (42.9)	8 (28.5)	0.725 ± 0.136
Femoral neck				
21–30	8 (80.0)	2 (20.0)	0 (0.0)	0.850 ± 0.135
31–40	31 (58.5)	17 (32.1)	5 (9.4)	0.876 ± 0.128
41–50	35 (70.0)	14 (28.0)	1 (2.0)	0.832 ± 0.122
51–60	29 (37.2)	30 (38.5)	19 (24.4)	0.786 ± 0.126
61–70	10 (35.7)	10 (35.7)	8 (28.6)	0.678 ± 0.127

Values are expressed as number (%) or mean ± standard deviation.

BMD, bone mineral density.

### 3.2 *Correlation coefficients of the texture analysis values and DXA-measured BMD*

**Fig. 3:**
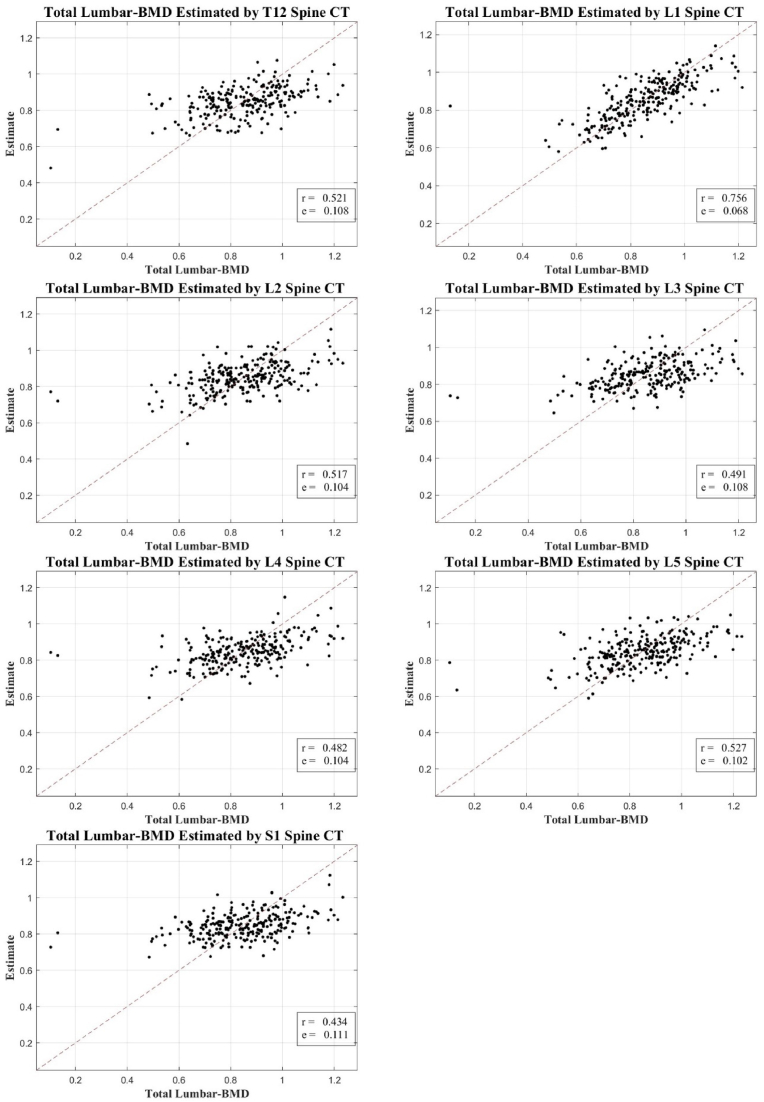
Correlation scatter plot: estimated BMD from T12-S1 CT axial cuts vs. total lumbar DXA BMD. BMD, bone mineral density; CT, computed tomography; DXA, dual-energy X-ray absorptiometry.

**Fig. 4:**
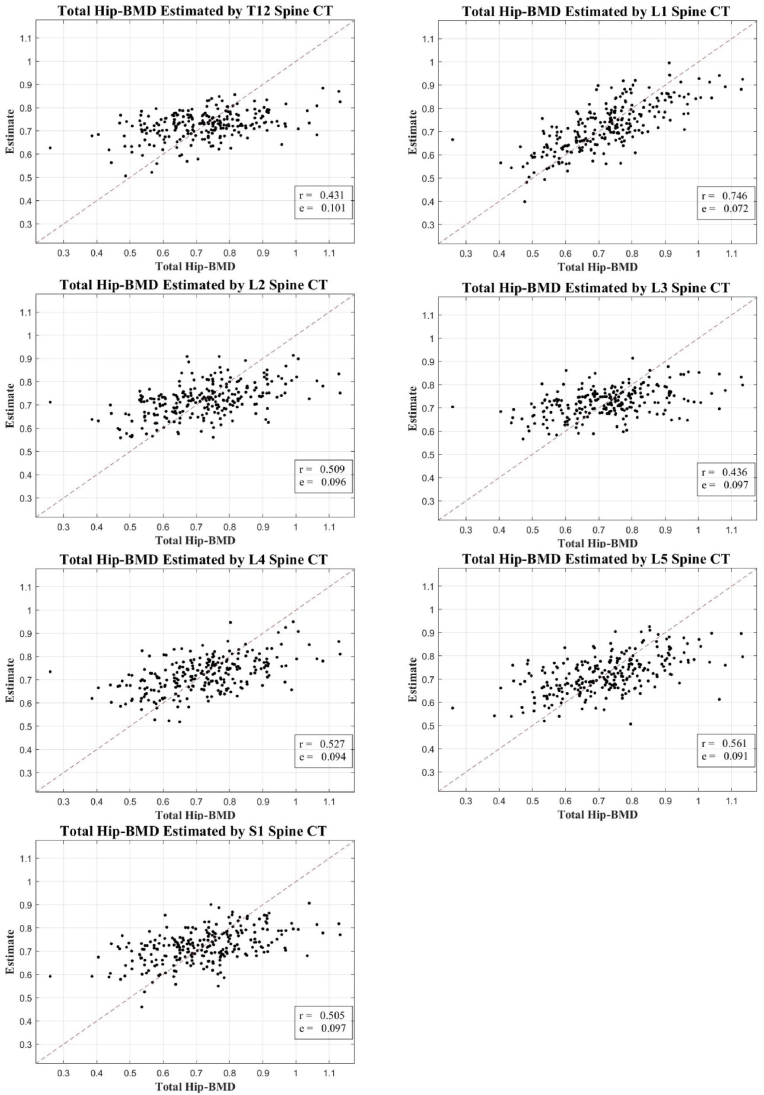
Correlation scatter plot: estimated BMD from T12-S1 CT axial cuts vs. total hip DXA BMD. BMD, bone mineral density; CT, computed tomography; DXA, dual-energy X-ray absorptiometry.

We observed a significant correlation between the texture analysis values derived from axial cuts of spinal CT scans and DXA-measured BMD. However, this correlation was modest, falling within the range of 0.431–0.561 for total hip BMD (excluding L1), and 0.434–0.527 for total lumbar BMD (excluding L1). These findings are depicted in Fig. 3, Fig. 4, using scatter plots to show the correlation between the estimated and actual DXA BMD measurements.

It's important to note that while our research explores the potential of spinal CT texture analysis as a diagnostic tool for bone health assessment, it does not position this method as a definitive standard for diagnosing osteoporosis. The observed correlation with DXA measurements, although significant, is modest and may reflect the inherent inaccuracies of DXA scanning, particularly in patients with degenerative changes or other factors that may compromise the accuracy of DXA.

This study represents an initial attempt to use a wider range of ROI (from T12 to S1) than typical DXA analysis (L1 to L4). Despite the modest correlation achieved, we believe this approach opens new possibilities for assessing bone health in cases where DXA scanning is not feasible or compromised.

## 4 Discussion

Our research focused on the potential of machine learning methodologies, specifically LR models [9], and texture analysis of Computed Tomography Hounsfield Units (CT HU), in the detection and assessment of osteoporosis. By extracting a broad set of features from spinal CT scans, we aimed to provide estimates of bone mineral density (BMD) that correlate with DXA measurements.

In our study of 405 cases from 219 patients, we demonstrated a significant, albeit modest, correlation between the CT-derived estimates and DXA-measured BMD. Notably, the highest correlation was observed in the L1 vertebral region, consistent with the DXA measurement location, indicating that the alignment of the estimation and measurement sites could enhance the correlation. However, the correlation for total hip and total lumbar BMD (excluding L1) was less pronounced, ranging between 0.431–0.561 and 0.434–0.527, respectively, suggesting the necessity for further refinement of the method.

Furthermore, we adopted a broader range of Regions of Interest (ROIs) from T12 to S1 for CT texture analysis, as opposed to the typical L1 to L4 range used in DXA. This opportunistic usage of the existing spinal CT scans not only leveraged an underutilized imaging resource but also widened the area of investigation, offering a potentially more comprehensive assessment of spinal bone health.

Interestingly, our study also revealed discordance between the CT and DXA measurements in certain scenarios. This discordance could stem from various factors, including differences in the measured regions, methodological discrepancies, or inherent inaccuracies in DXA scanning, particularly in patients with degenerative changes or abdominal obesity [10]. DXA measurements are known to be susceptible to inaccuracies in individuals with high abdominal fat content due to overlaying soft tissue artifacts [3]. On the contrary, spinal CT, particularly when encompassing a broader ROI, might provide a more accurate reflection of the bone health in these patients.

Our research showcases the advantageous usage of CT scans in estimating BMD, especially in the context of increased overall obesity. Unlike DXA-estimated BMD, CT scans are less affected by surrounding adipose layers and soft tissue inhomogeneity, thereby minimizing the “noise" in the BMD estimation.

We chose to employ spine CT scans for this texture analysis due to their frequent usage during health checkups, often alongside DXA scans for osteoporosis diagnosis. These scans encompass regions of interest (T12-S1), which are integral to DXA BMD measurements. Moreover, CT HU measurement provides a straightforward representation of BMD using the tissue density of vertebral trabecular bone mass. To further refine this process, we utilized the Gray Level Co-Occurrence Matrix (GLCM), a widespread method in texture analysis. Extracted statistical parameters from GLCM, including energy, contrast, entropy, and others, facilitate a quantitative understanding of the spatial relationship between pixels in the analyzed area [11].

In contrast to previous studies, our investigation is not limited to the L1 region [12]. Instead, we considered a broader range, spanning from T12 to S1, a factor that enhances the robustness of our findings. However, our study still carries certain limitations, such as a relatively small sample size from a single center and a lack of consideration for aortic calcification. Nevertheless, these constraints do not overshadow our primary finding: the value of CT scans for BMD estimation, particularly considering their potential in screening patients for osteoporosis risk without additional diagnostic tests. While further research is necessary to consolidate these insights, our study signals a promising direction in addressing discordance between spine CT texture analysis and DXA BMD.

Although our research is a significant stride towards integrating machine learning with radiomics for osteoporosis assessment, it also highlights the need for further research in this area. Given the modest correlations achieved and the discordance observed with DXA measurements, CT-derived BMD estimates may not yet be ready to serve as a standard diagnostic tool for osteoporosis. However, they do offer an alternative perspective, which, in conjunction with traditional methods like DXA, could potentially provide a more holistic and accurate assessment of bone health. In addition, a notable limitation of our study is the potential for selection bias due to the inclusion of multiple images from the same patients, particularly those under follow-up. This aspect of our study design was essential for observing the temporal changes in texture analysis features and DXA-based BMD values over time. However, it is important to acknowledge that this approach might have introduced a bias by overrepresenting certain patient profiles. Future studies could benefit from a more diversified patient pool to mitigate this limitation and provide a more comprehensive understanding of the disease progression in a wider patient demographic. We believe this acknowledgment of potential bias adds to the transparency and rigor of our research findings. Also, our study's limitation lies in not accounting for comorbid conditions like renal dysfunction, diabetes, and obesity, which can affect bone quality. The lack of this data may limit the applicability of our findings across varied patient profiles. Recognizing these factors is essential for a comprehensive understanding of bone health in future studies.

In summary, our study underscores the potential of CT HU texture analysis and machine learning models in providing a new lens for osteoporosis detection and monitoring. Despite certain limitations, this approach offers a promising starting point for future studies aiming to leverage the underutilized resource of spinal CT scans in bone health assessment. Further investigations involving larger and more diverse patient groups, and refined methodologies, could potentially corroborate and expand upon our findings.

## 5 Conclusions

Emerging from this study is a novel insight into the discordance between spinal CT texture analysis and DXA derived BMD measurements, highlighting the unique influence of spinal attributes. This revelation calls into question the exclusive reliance on DXA scans for BMD assessment, particularly in scenarios where DXA scanning may not be feasible or accurate.

## CRediT author statement

**Min Woo Kim:** Conceptualization, Validation, Resources, Writing—Review and editing, Project administration. **Young Min Noh:** Software, Supervision. **Jung Wook Huh:** Methodology, Visualization. **Han Eol Seo:** Validation. **Dong Ha Lee:** Conceptualization, Validation, Formal analysis, Investigation, Data curation, Writing—Original draft.

## Conflicts of interest

The authors declare no competing interests.
